# An Energy Balanced and Lifetime Extended Routing Protocol for Underwater Sensor Networks

**DOI:** 10.3390/s18051596

**Published:** 2018-05-17

**Authors:** Hao Wang, Shilian Wang, Eryang Zhang, Luxi Lu

**Affiliations:** 1 College of Electronic Science and Engineering, National University of Defense Technology, Changsha 410073, China; wanghao08@nudt.edu.cn (H.W.); eryangzh@163.com (E.Z.); 2 National Key Laboratory of Science and Technology on Blind Signal Processing, Chengdu 610000, China; luluxi@pku.edu.cn

**Keywords:** underwater communication, energy consumption, energy efficiency, routing protocols, network lifetime

## Abstract

Energy limitation is an adverse problem in designing routing protocols for underwater sensor networks (UWSNs). To prolong the network lifetime with limited battery power, an energy balanced and efficient routing protocol, called energy balanced and lifetime extended routing protocol (EBLE), is proposed in this paper. The proposed EBLE not only balances traffic loads according to the residual energy, but also optimizes data transmissions by selecting low-cost paths. Two phases are operated in the EBLE data transmission process: (1) candidate forwarding set selection phase and (2) data transmission phase. In candidate forwarding set selection phase, nodes update candidate forwarding nodes by broadcasting the position and residual energy level information. The cost value of available nodes is calculated and stored in each sensor node. Then in data transmission phase, high residual energy and relatively low-cost paths are selected based on the cost function and residual energy level information. We also introduce detailed analysis of optimal energy consumption in UWSNs. Numerical simulation results on a variety of node distributions and data load distributions prove that EBLE outperforms other routing protocols (BTM, BEAR and direct transmission) in terms of network lifetime and energy efficiency.

## 1. Introduction

Recent advances in underwater sensor networks (UWSNs) have motivated the development of various applications for scientific, environmental, commercial and military purposes including environmental data collection, disasters prevention, assisted navigation, monitoring underwater equipments, offshore exploration, oil/gas spills monitoring and tactical surveillance [1,2,3,4]. To avoid the high absorption rate of electromagnetic waves and scattering of optical waves in water, acoustic waves are preferred for long-distance underwater communication. However, adverse characteristics of UWSNs [5,6,7] such as dynamic structure, high energy consumption, limited available bandwidth, limited battery power, low transmission speed, severely attenuated channel, high latency, and high bit error rates pose great challenges to reliable underwater data transmissions.

In order to prolong the network lifetime, some researchers focus on the energy harvesting technique [8,9,10] so that the sensor nodes can harvest energy from the environment and solve the energy limitation problem. However, the underwater sensors cannot use solar power chargers as it is hard for the sunlight to reach the deep sensors in an underwater environment. Besides, the underwater sensors are vulnerable to the seawater corrosion and marine animals’ activities. The energy harvesting technique still needs to be improved in underwater environments.

Multi-hop transmission is a promising technique for decreasing energy consumption and enhancing system stability in UWSNs and many underwater applications such as environmental data collection (temperature, conductivity, pH, dissolved oxygen, etc.), imaging underwater life, supervising geological processes on the ocean floor, and monitoring underwater equipments exploiting multi-hop communication to collect sensed data and forward them to the sink nodes on the water surface. So the routing protocol, which aims at choosing a reliable and energy efficient path to forward data to the gateway nodes, is essential in underwater data transmissions. As it is hard to replace the battery in underwater nodes, energy limitation is a vital problem in underwater routing protocol design.

Recently, many energy efficient routing protocols for UWSNs were proposed [11,12,13,14,15]. These energy efficient routing protocols may consume less energy in total, but the energy consumption may focus on a few hot spots and it makes these nodes deplete their energy at an earlier time while other nodes may still have a lot of energy left. This unhealthy energy distribution leads to the void region problem and should be avoided. To solve this problem, a energy balancing technique [16,17] is proposed to balance the energy consumption in UWSNs. However, these balanced energy routing protocols induce frequent long range direct transmissions which result in high energy consumption and high probabilities of packet collision problem. In some large scale UWSNs, long range direct transmissions are unable to be realized due to the transmission power limitations of sensor nodes.

**Figure 1:**
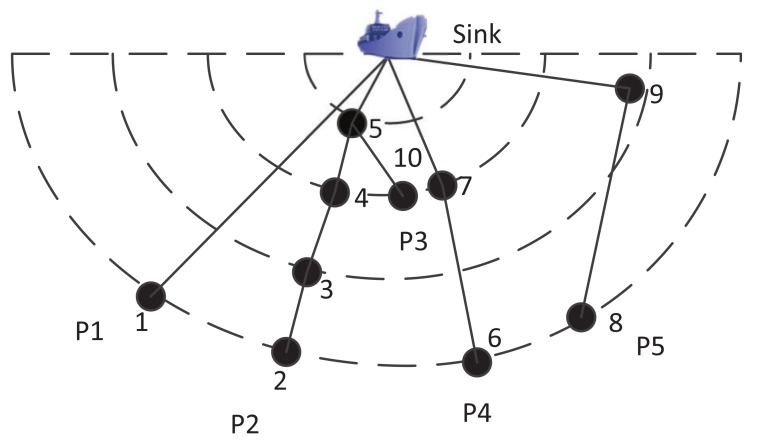
Explanations on the basic idea of energy balanced and lifetime extended routing protocol (EBLE).

In this paper, an energy balanced and lifetime extended protocol (EBLE) is proposed to prolong the underwater network lifetime. The routing process is divided into two phases: (1) candidate forwarding set selection phase and (2) data transmission phase. In candidate forwarding set selection phase, each node stores the position and residual energy information of its neighborhood nodes and obtains the cost values according to our proposed cost function. During the data transmission phase, high residual energy level and low cost nodes are given higher priorities to forward data and then long range direct transmissions are avoided whereas the network maintains energy balance. A brief explanation of the basic idea of EBLE is shown in Figure 1. A sink node is located on the surface and several sensor nodes are deployed in the semicircle around the sink. There are five paths P1∼P5 (solid lines) to transmit sensed data to the sink via multi-hop communications. The power consumption increases rapidly with the increase of communication range (proved in Section 4.1), so the power consumption of P1 is much higher than P2 and P4, and data transmissions in P4 consume more energy power than P2. In order to save energy, P2 is preferred when all nodes have enough residual energy. However, as the traffic load of nodes nearby the sink (e.g., node 5) is much higher than that of nodes far away from the sink, a few nodes may deplete their energy while other nodes have abundant residual energy. This unbalanced energy consumption may result in void region problems in UWSNs and sensed data in some regions cannot be forwarded to the sink node efficiently. Some existing energy efficient protocols [11,12,13,14,15] only focus on finding energy efficient paths, which may easily lead to void region problems. Energy balancing protocols like BEAR [16] and BTM [17] change their transmission mode to direct one-hop transmissions if their optimal energy efficient paths are energy limited. This operation introduces extra energy consumption and cannot use low traffic load nodes efficiently. To prolong the network lifetime, some low traffic load nodes (e.g., node 10) should be distributed with more loads. These low traffic load nodes can communicate with some high residual energy nodes (perhaps with long communication ranges) and relax the burden of other low residual energy nodes. For example, when node 7 lacks energy, node 6 should select node 10 to relay data while current energy balancing protocols like BTM just allow node 6 to transmit data directly to the sink. Another aspect that should be taken into consideration is energy efficiency optimization. Too long distance communication can consume the energy power at a high speed and thus the network lifetime is reduced. In Figure 1, path P1 consumes more power than path P4, but consumes less energy than P5. So path P5 is only chosen when other paths are lacking residual energy obviously or there are no other paths. In our proposed protocol, the aim is to choose both energy balanced and energy efficient paths to forward data packets. The main contributions in this paper are summarized as follows:

- An energy balanced and lifetime extended routing protocol, EBLE, is proposed. EBLE can choose several successor nodes according to the cost function and residual energy. When a possible forwarder’s energy level is lower than the sender, another suboptimal forwarder can take the place of the current one and thus the energy consumption is more balanced. The network lifetime is extended through choosing both energy balanced and efficient routes.
- Detailed analysis of optimal energy consumption for different transmission modes are given and an energy cost function is proposed to optimize data transmissions in UWSNs.
- Extensive simulations are conducted to verify the effectiveness and validity of our proposed EBLE and the results show that EBLE outperforms other existing energy balancing routing protocols in terms of energy consumption and network lifetime. Using EBLE, more packets can be transmitted before a first node in the network is depleted of its battery power. The network lifetime is prolonged by 62.8% on average when compared with BTM on random node distribution case.

The rest of the paper is organized as follows: Section 2 shows the recent hot research topics and the related research of UWSNs routing protocols. Section 3 describes the network and energy models in UWSNs and makes some assumptions about our proposed protocol. The detailed design of EBLE is illustrated in Section 4 and numerous simulations are given in Section 5. Section 6 concludes the paper at last.

## 2. Related Work

Energy limitation, unstable links, long end-to-end delay are key characteristics of UWSNs. Therefore, numerous UWSNs routing protocols have been devoted to solving these problems. In this section, we discuss some of the existing UWSNs routing protocols and analyze their limitations in dealing with underwater reliable transmissions.

Peng Xie et al. [13] proposed a vector-based forwarding protocol (VBF) for underwater sensor networks. VBF is a position-based routing approach and a routing “pipe” path is constructed to guide data transmissions, only nodes close to the vector from the source to the destination are chosen to forward the message. In this way, only a small fraction of the nodes are involved in routing. VBF also adopts a self-adaptation algorithm which allows nodes to weigh the benefit of forwarding packets. The data forwarding process in low priority nodes can be suppressed to avoid too many redundant transmissions. Packet delivery ratio and average delay performance are improved at the cost of energy consumption. HHVBF [18] was later proposed to further improve the packet delivery ratio performance and robustness of VBF. HHVBF uses hop-by-hop routing vectors and is less sensitive to “routing pipe” radius threshold. Results show that HHVBF can improve data delivery ratio performance for sparse networks but consumes more energy. DFR [12] exploits a packet flooding technique to increase the reliability. The number of forwarding packets nodes is controlled with the information of node position and link quality. DFR also performs well in data delivery ratio at the cost of large and unbalanced energy consumption.

In [11], a depth-based routing (DBR) protocol was proposed to forward data from the seafloor to the sea surface. A key advantage of DBR is that DBR does not require full-dimensional location information of sensor nodes and only needs nodes depth information which can be easily obtained with an inexpensive depth sensor. DBR can achieve high packet delivery ratios (at least 95%) for dense networks but multiple sinks are required and redundant transmissions are induced. EEDBR [19] is an enhanced version of DBR. Different from DBR, the residual energy of the sensor nodes in EEDBR is also taken into account to improve the network life-time and energy consumption is balanced to some extent. An energy-efficient and void avoidance depth based routing (EVA-DBR) [20] protocol was proposed which can exclude trapped and void nodes from the routing paths using a passive participation approach. The number of participated nodes can be adjusted by changing transmission range settings and then redundant transmissions can be controlled.

Youngtae Noh et al. [15] proposed a void-aware pressure routing protocol (VAPR) which uses periodic beacons to set up next-hop directions and to build a directional trail to the closest sonobuoy. The hydraulic pressure based anycast routing [21] (HydroCast) also utilizes periodic beacons to obtain the neighbor nodes and prioritize forwarding nodes with Expected Packet Advance (EPA). VAPR and HydroCast can solve the void region problem but node residual energy is not considered and some nodes may deplete their energy due to the unbalanced heavy traffic load.

Flooding-based protocols perform well in terms of packet delivery ratio in underwater sensor networks but are usually not energy efficient. To solve the high energy consumption problem, Elvin Isufi et al. [22] proposed an advanced flooding-based routing protocol for UWSNs. The number of the replicas can be reduced by considering the relative positions to reduce the number of relays. The selection of relays is based on the relative distance between relay and source/destination. A node is only chosen as a candidate forwarder when it is located inside the communication range of the source and is enough close to the destination. Moreover, network coding is introduced into transmissions. The relay nodes in the network can cancel duplicated transmissions by checking whether the received packet is an innovative one or not. Only independent packets can be encoded and forwarded to the sink. This scheme outperforms other flooding-based routing protocols in terms of energy consumption.

In [5], a channel-aware routing protocol called CARP was proposed to select robust links with link quality information. CARP uses a PING-PONG strategy to obtain the adjacent nodes information. Channel quality, residual energy and buffer space are also considered for selecting best relays. E-CARP [23] improves CARP by considering the reusability of previously collected sensory data. The sensory data are required to be routed to a relay node, only when the difference between the current data and the previous one is obvious enough. Besides, the PING-PONG strategy is simplified and the route table remains unchanged if the network topology is relatively steady.

Recently, a group of balanced energy consumption routing protocols for UWSNs were proposed to balance the energy consumption in the network and then prolong the network life. Jinfeng Dou et al. presented a probability and sub-optimal distance-based lifetime prolonging strategy (PAS) [24] for UWSNs. Energy consumption is balanced by carefully choosing transmission modes between single-hop direct transmission (DT) and multi-hop transmission (MT). The network is divided into many circular slices. Then a probability finding algorithm (PFA) can find a set of probabilities for each slice to decide the nodes’ transmission mode.

A similar balance transmission mechanism called BTM [17] was proposed by Jiabao Cao et al. BTM divides the transmission process into two phases. Nodes operate an efficient routing algorithm in the routing set-up phase. In the stable data transmission phase, nodes determine one-hop or multi-hop data transmissions based on the adjacent nodes energy level. If the adjacent node energy level is lower than the energy level of the forwarding node, one-hop direct data transmission to the sink is performed. Or else, data can be forwarded to the sink via multi-hop transmissions. This scheme can balance the energy consumption between two adjacent nodes. However, each node only has one relay node and unbalanced transmissions still exist if nodes and traffic load are distributed unevenly.

Javaid et al. proposed a balanced energy consumption based adaptive routing [16] (BEAR) scheme for IoT enabling underwater WSNs. In BEAR, the network field is divided into a series of cones, the traffic load can be evenly distributed by using zone-to-zone communications. Moreover, BEAR generally chooses two alternative relay nodes. One-hop direct transmissions are only carried out when the residual energy of the two alternative relay nodes are both lower than the average residual energy of the network. Results show that BEAR can prolong the network lifetime significantly.

Hanjiang Luo et al. presented two energy balanced strategies [25] to maximize the lifetime of networks. The first is an energy balanced hybrid data propagation algorithm (EBH) which changes the transmission mode between multi-hop and one-hop transmissions according to the residual energy grade. The second is differential initial battery assignment strategy which tries to pre-assign differential initial battery power according to workloads in different nodes.

Although the above balance transmission mechanism can balance the energy consumption between different traffic load nodes and prolong the network lifetime to some extent, a great amount of energy is wasted due to the long range one-hop transmissions that balance transmission mechanism brings. Moreover, some UWSNs applications cannot bear such long range transmissions because of the heavy packet collisions and high requirements of the power amplifier. Therefore, their applications are limited in practical use.

## 3. Models and Assumptions

In this section, we present the network model and channel model and then give some assumptions of our proposed protocol. The energy consumption is also derived in this section.

### 3.1. Network Model

**Figure 2:**
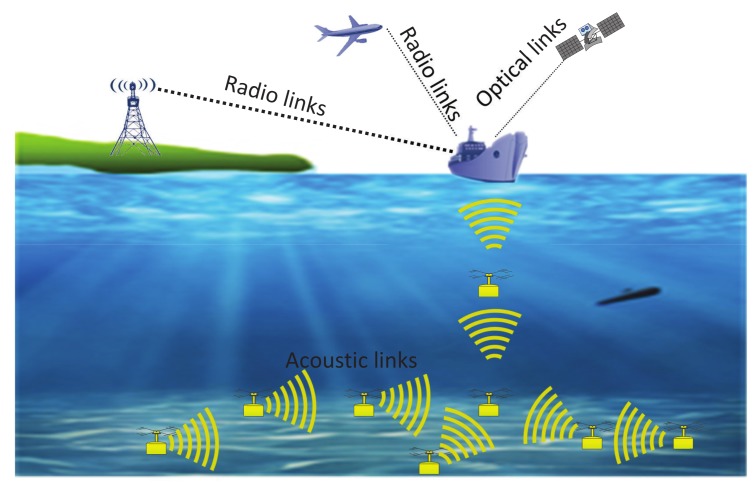
Network model of underwater sensor networks (UWSNs).

The network model of UWSNs is illustrated in Figure 2. Several sensor nodes are clustered and distributed randomly in the underwater environment. These sensor nodes are anchored to the ocean bottom and construct a quasi-stationary architecture [26]. Acoustic communications are used for sensing and forwarding data to the sink node. Nodes far away from the sink node can use multi-hop communication to transmit data. The sink node, usually located in the center among these sensor nodes, collects data from those sensor nodes via horizontal acoustic links. Then data are stored and further forwarded to the shore-based stations or moving platforms by vertical acoustic links. We assume that the packets sent by the sensor nodes are delivered successfully if they reach the sink node.

### 3.2. Channel and Energy Consumption Model

The energy consumption is affected by the underwater channel. Here we present the description of path loss and ambient noise of underwater channel and derive the energy consumption model for UWSNs. Urick gave an empirical formula in 1967 [27] to describe the underwater path loss and attenuation as in (1).

(1) $$A\left(d,f\right)=A_{0}d^{k}a{\left(f\right)}^{d}$$

$A\left(d,f\right)$ is the path loss for a given distance *d* and frequency *f*. $A_{0}$ is a unit-normalizing constant. *k* is the spreading factor and k=2 is for spherical spreading in deep sea communications. a(f) is the absorption coefficient. The path loss can be expressed as TL in dB [28] by (2).

(2) $$TL=10\log A\left(d,f\right)/A_{0}=k\cdot 10\log d+d\cdot 10\log a\left(f\right)$$

Then 10loga(f) is given by Thorp [29] in dB/km for *f* in kHz as in (3).

(3) $$\begin{array}{cc} 10\log a(f)= & 0.11\frac{f^{2}}{1+f^{2}}+44\frac{f^{2}}{4100+f^{2}}\\ & +2.75\times 10^{-4}f^{2}+0.003,f>200\mathrm{Hz} \end{array}$$

The underwater ambient noise is modeled by Wenz [6] as in (4).

(4) $$NL\left(f\right)=N_{t}\left(f\right)+N_{s}\left(f\right)+N_{w}\left(f\right)+N_{th}\left(f\right)$$

$N_{t}\left(f\right)$, $N_{s}\left(f\right)$, $N_{w}\left(f\right)$, and $N_{th}\left(f\right)$ represent the effect of turbulence, shipping, waves, and thermal noise at a given frequency *f*, respectively. The total ambient noise NL(f) is the sum of the four parts. These four parts can be obtained from [6,28]. Then the signal-to-noise ratio (SNR) can be derived according to the passive sonar equation [27] as in (5).

(5) $$\gamma_{b}=SL-TL-NL+DI\geq DT$$

$\gamma_{b}$ is the SNR at the receiver and DI is the directive coefficient. For omnidirectional hydrophones, DI=0. DT is the minimal SNR required for signal acquisition at the receiver. SL is the output acoustic source level in dB re μPa at the sender and the reference value of 1 μPa equals to $0.67\times 10^{-18}$ Watts/m${}^{2}$ [25]. Derived from (5), we can obtain the minimal SL at the sender as in (6).

(6) $$SL=DT+TL+NL-DI=10\log\frac{I_{T}}{0.67\times 10^{-18}}$$

Here $I_{T}$ is the transmitted signal intensity at 1 m in Watts/m${}^{2}$ from the source. For spherical spreading, the transmit power $P_{tx}$ at the sender can be derived from (7).

(7) $$P_{tx}=4\pi \times {\left(1m\right)}^{2}\times I_{T}$$

From (6) and (7), the transmit power can be expressed as in (8).

(8) $$P_{tx}=4\pi \times 0.67\times 10^{-18}\times 10^{(DT+TL+NL-DI)/10}$$

Suppose that there are $N_{tx}$ nodes transmitting data, $N_{rx}$ nodes receiving data, and $N_{idle}$ nodes staying idle in the network within a short time *t*. The total energy consumption of the network in *t* can be expressed as in (9).

(9) $$\begin{array}{cc} E_{t} & =E_{tx}+E_{rx}+E_{idle}\\ & =t\left(\sum_{i=1}^{N_{tx}}P_{tx}\left(i\right)+\sum_{j=1}^{N_{rx}}P_{rx}+\sum_{k=1}^{N_{idle}}P_{idle}\right) \end{array}$$

Here $E_{tx}$, $E_{rx}$, and $E_{idle}$ denote the energy consumption in transmitting, receiving, and idle state, respectively. $P_{tx}\left(i\right)$ is the transmit power for node *i*. $P_{rx}$ and $P_{idle}$ are the receiving and idle power in Watts for one node. We assume that all sensor nodes are homogeneous, so $P_{rx}$ and $P_{idle}$ are the same for each node. Our aim is to minimize the total energy consumption before some nodes in the network deplete their energy as in (10).

(10) $$\left\{\begin{array}{c} \min\left(\int_{0}^{T_{d}}E_{t}dt\right)\\ \exists i\in \left[1,N\right]\;s.t.\;E_{res}\left(i,T_{d}\right)=0 \end{array}\right.$$

*N* is the total number of sensor nodes in the network. $T_{d}$ is the time when a first node is depleted of its battery power. $E_{res}\left(i,T_{d}\right)$ is the residual energy of node *i* at time $T_{d}$. Note that the sink node can usually collect energy from solar power, so the sink node is considered to have infinite energy and the energy consumption at sink node is not considered in protocol design.

### 3.3. Assumptions

We make the following assumptions in this part.

1. All sensor nodes are homogeneous but deployed randomly around the sink node on the sea floor. These nodes rarely move after deployment. The data packets are considered to be transmitted successfully when they reach the sink node. The sink node is equipped with more battery power or can be recharged though energy harvesting. So the sink node can keep on working until all sensor nodes are dead.
2. Each sensor node is equipped with an adaptive power control module. That is to say, all sensor nodes can adjust the transmit power according to the expected transmission range. The maximum transmit power and the maximum transmission range of sensor nodes are identical. The sink node is within the transmission range of the farthest sensor node. The signal processing power is negligible when compared with data transmission power.
3. The sensor nodes may not be aware of their own location, but they can obtain the relative distance to their neighborhood nodes by measuring the Received Signal Strength Indicator (RSSI) of the received signal. The technique is widely used in underwater sensor networks and this assumption is justified by the fact that acoustic directional antennae are of much smaller size than RF directional antennae due to the extremely small wavelength of sound. Moreover, underwater sensor nodes are usually larger than land-based sensors, and they have room for such devices [13].
4. Proper medium access control methods (e.g., CDMA-based or slotted contention window based technologies [30]) can be used to achieve multiple simultaneous wireless transmissions.

These assumptions are reasonable due to the development of underwater modems and other acoustic communication hardware.

## 4. Energy Balanced and Lifetime Extended Protocol (EBLE)

In this section, we present the problem description and our proposed EBLE protocol in detail.

### 4.1. Problem Description

**Figure 3:**
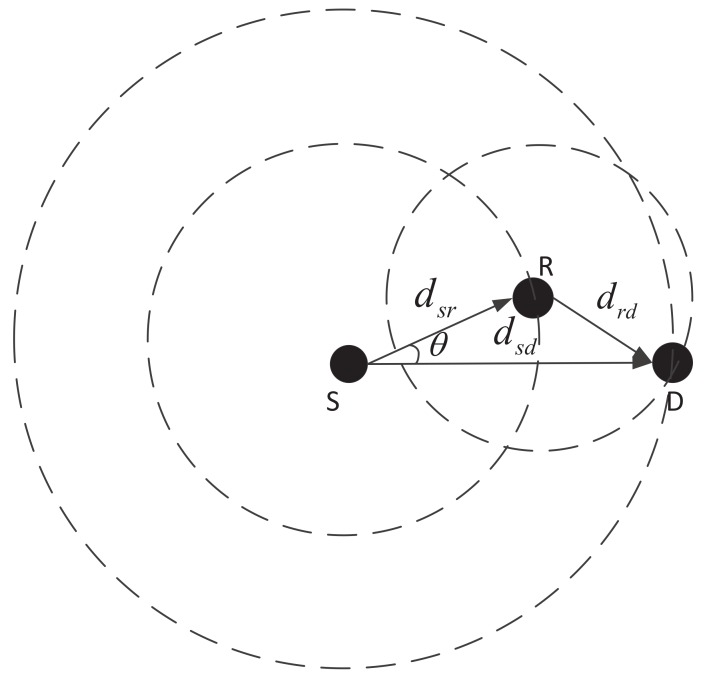
A simple transmission case in UWSNs.

We analyze the effect of energy balancing and energy efficiency in different data transmission cases firstly. Then we present some optimal principles for designing energy balanced and efficient routing protocol. We suppose the following transmission case as shown in Figure 3: one relay node *R* is deployed between the source node *S* and the destination node *D*. The distances between source and destination, source and relay, relay and destination are $d_{SD}$, $d_{SR}$, and $d_{RD}$, respectively. θ is the angle between vector $\vec{SR}$ and vector $\vec{SD}$. We analyze the energy consumption of one-hop direct transmission and multi-hop transmission via the relay node.

**Theorem** **1.** *When the relay node is deployed on the straight line between the source and the destination (θ=0), multi-hop transmissions are more energy efficient than one-hop direct transmission if we neglect the receiving power.*

**Proof of** **Theorem 1.** As DT, NL and DI are transmission range independent, we rewrite the transmit power as in (11).

(11) $$\begin{array}{cc} P_{tx} & =P_{0}10^{TL/10}\\ & =P_{0}d^{k}a{\left(f\right)}^{d} \end{array}$$

where $P_{0}$ is a distance independent parameter and can be expressed as in (12).

(12) $$P_{0}=4\pi \times 0.67\times 10^{-18}\times 10^{(DT+NL-DI)/10}$$

Suppose that the packet length is *L* bits and the data rate is Ra bits/s. The energy consumption of transmitting one packet to distance *d* can be expressed in (13).

(13) $$\begin{array}{cc} E_{tx}\left(d\right) & =\left(L/Ra\right)\left(P_{tx}+P_{rx}\right)\\ & =\frac{LP_{0}d^{k}a{\left(f\right)}^{d}}{Ra}+\frac{LP_{rx}}{Ra} \end{array}$$

Here $P_{rx}$ is the receiving power of the receiver. We assume that the relay and the sender can overhear the data packet of each other when they are located inside the transmission range. Then energy consumption for one-hop direct transmission $E_{direct}$ can be expressed as in (14).

(14) $$E_{direct}=\frac{LP_{0}d_{SD}^{k}a{\left(f\right)}^{d_{SD}}}{Ra}+2\frac{LP_{rx}}{Ra}$$

Similarly, the energy consumption of multi-hop transmission $E_{multi-hop}$ can be obtained from (15).

(15) $$E_{multi-hop}=\frac{LP_{0}}{Ra}\left(d_{SR}^{k}a{\left(f\right)}^{d_{SR}}+d_{RD}^{k}a{\left(f\right)}^{d_{RD}}\right)+m\frac{LP_{rx}}{Ra}$$

Here *m* is the number of possible receivers and m=2 for $d_{SR}>d_{RD}$, m=3 for $d_{SR}\leq d_{RD}$. As the relay node is deployed right on the straight line between the source and destination. So $d_{RD}+d_{SR}=d_{SD}$ and we can rewrite (15) as in (16).

(16) $$\begin{array}{c} E_{multi-hop}=\frac{LP_{0}}{Ra}d_{SR}^{k}a{\left(f\right)}^{d_{SR}}+\frac{LP_{0}}{Ra}{\left(d_{SD}-d_{SR}\right)}^{k}a{\left(f\right)}^{d_{SD}-d_{SR}}+m\frac{LP_{rx}}{Ra} \end{array}$$

In order to minimize $E_{multi-hop}$, we can obtain the derivation of $E_{multi-hop}$ with respect to $d_{SR}$ as in (17).

(17) $$\begin{array}{cc} E_{multi-hop}^{\prime }= & \frac{LP_{0}}{Ra}\{d_{SR}^{k-1}a{\left(f\right)}^{d_{SR}}\left(k+d_{SR}\ln a\left(f\right)\right)\\ & -k{\left(d_{SD}-d_{SR}\right)}^{k-1}a{\left(f\right)}^{d_{SD}-d_{SR}}\\ & -{\left(d_{SD}-d_{SR}\right)}^{k}a{\left(f\right)}^{d_{SD}-d_{SR}}\ln a\left(f\right)\} \end{array}$$

When $E_{multi-hop}^{\prime }=0$, we can have $d_{RD}=d_{SR}=d_{SD}/2$ and the minimal value and maximum value of $E_{multi-hop}$ is shown in (18) and (19).

(18) $$\begin{array}{c} \min\left(E_{multi-hop}\right)=\frac{LP_{0}}{Ra2^{k-1}}\left(d_{SD}^{k}a{\left(f\right)}^{d_{SD}/2}\right)+m\frac{LP_{rx}}{Ra} \end{array}$$

(19) $$\max\left(E_{multi-hop}\right)=E_{direct}+\left(m-2\right)\frac{LP_{rx}}{Ra}$$

As the receiving power is much lower than the transmitting power [31]. So we can roughly believe $\max\left(E_{multi-hop}\right)=E_{direct}$. So it has been proved that the multi-hop transmission mode is more energy efficient in this case. ☐

Next we consider a general case in which the relay node is just deployed between the source and the destination (θ∈[−π/2,π/2]).

**Theorem** **2.** *If there is only one relay node, multi-hop transmission is no longer energy efficient when $d_{SR}^{k}a{\left(f\right)}^{d_{SR}}+d_{RD}^{k}a{\left(f\right)}^{d_{RD}}>d_{SD}^{k}a{\left(f\right)}^{d_{SD}}$.*

**Proof of** **Theorem 2.** From Equations (14) and (15) and we set $E_{multi-hop}>E_{direct}$. We can obtain (20)

(20) $$\frac{LP_{0}}{Ra}\left(d_{SR}^{k}a{\left(f\right)}^{d_{SR}}+d_{RD}^{k}a{\left(f\right)}^{d_{RD}}-d_{SD}^{k}a{\left(f\right)}^{d_{SD}}\right)+\left(m-2\right)\frac{LP_{rx}}{Ra}>0$$

As m≥2, the inequality is always reasonable when $d_{SR}^{k}a{\left(f\right)}^{d_{SR}}+d_{RD}^{k}a{\left(f\right)}^{d_{RD}}>d_{SD}^{k}a{\left(f\right)}^{d_{SD}}$. So it is proved that if there is only one relay node, multi-hop transmission is no longer energy efficient when $d_{SR}^{k}a{\left(f\right)}^{d_{SR}}+d_{RD}^{k}a{\left(f\right)}^{d_{RD}}>d_{SD}^{k}a{\left(f\right)}^{d_{SD}}$. ☐

**Figure 4:**
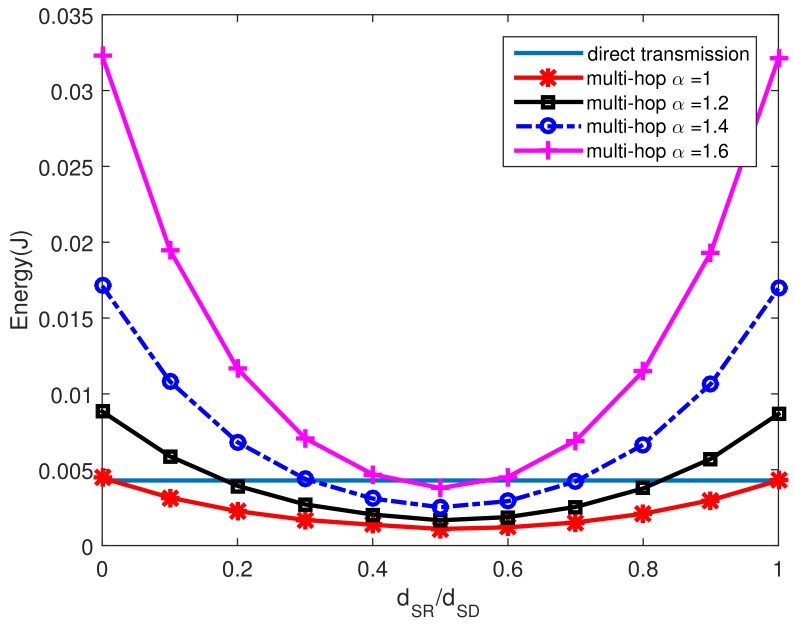
Energy consumption for different α.

To further explain the energy consumption in UWSNs, we assume that $d_{SR}+d_{RD}=\alpha d_{SD}$. Figure 4 illustrates the energy consumption for one-hop direct transmission and multi-hop transmissions with different α. The results show that the multi-hop transmission mode is often more energy efficient than one-hop direct transmission especially when the relay node is close to the middle position between the source and the destination. However, when $d_{RD}+d_{SR}$ is too large, the multi-hop transmission scheme is not energy efficient any more no matter where the relay node is. So we need to consider the relative distance to the source and destination when choosing forwarding nodes and designing the cost function. The detailed design for choosing the next forwarding nodes is illustrated in Section 4.2.

### 4.2. Detailed Design of EBLE

In this section, we present our design of EBLE in detail. The EBLE protocol operates in two phases: candidate forwarding set selection phase and data transmission phase.

#### 4.2.1. Candidate Forwarding Set Selection Phase

In this phase, the sink node broadcasts an indicator signal first so that each node can obtain its relative distance towards the sink by calculating the received signal strength. Then each sensor node broadcasts a packet to inform its neighbors of its relative distance towards the sink and its current residual energy level (EL). EL is defined as in [17], that is, we divide a node’s initial energy into *m* equal parts and the residual energy is larger than EL parts and smaller than EL+1 parts. The optimal EL division can be obtained from [17]. After the successful reception of broadcasting packets, each sensor node stores the EL of its neighbors and calculates the cost value $Q_{i,j}$ according to the cost function. The cost function for node *i* is described in (21).

(21) $$\begin{array}{cc} Q_{i,j} & =\frac{d_{c}^{k}{\left(a(f)\right)}^{d_{c}}}{d_{i,sink}-d_{j,sink}}\\ d_{c} & =\max(d_{i,j},d_{min}) \end{array}$$

Where $d_{i,j}$ is the distance between node *i* and node *j*, $d_{min}$ is the minimal transmission range. When $d_{i,j}<d_{min}$, the transmitting power of the transducers keeps unchanged at $P_{txmin}$. $d_{i,sink}$ is the distance between node *i* and the sink, and $d_{j,sink}$ is the distance between node *j* and the sink. The denominator, $\left(d(i,sink)-d(j,sink)\right)$, denotes the effective propagation distance towards the sink. The numerator reflects the energy consumption ratio to forward the data packet to node *j*. So the cost function can reflect the energy consumption per effective transmission distance. The aim of cost function is to select next hop forwarder with relatively long effective propagation distance and short actual propagation distance. So energy balancing and energy efficiency are both considered in our design. The node *j* would have a higher priority to be chosen as the next hop forwarder of node *i* if the value of $Q_{i,j}$ is smaller. In order to further reduce inefficient transmissions, node *j* is considered to be a candidate forwarder of node *i* only when the following conditions are met: (1) $d_{i,sink}>d_{i,j}$; (2) $d_{i,sink}>d_{j,sink}$; (3) $d_{i,j}^{k}a{\left(f\right)}^{d_{i,j}}+d_{j,sink}^{k}a{\left(f\right)}^{d_{j,sink}}<d_{i,sink}^{k}a{\left(f\right)}^{d_{i,sink}}$. The overall process of candidate forwarding set selection is shown in Algorithm 1. The relative parameters are defined in Table 1.
**Algorithm 1** Candidate Forwarding Set Selection1:**procedure**BroadcastPackets2:    Packet.AddHeader(NodePosition);3:    Packet.AddHeader(NodeEL);4:    Packet.SetPacketType(PacketType);5:    broadcast the packet;6:**end procedure**7: 8:**procedure**ReceivePackets9:    **if** Receive signal from sink **then**10:        $d_{i,sink}\leftarrow CalculateRelativeDistance\left(\right)$;11:    **else**12:        $d_{j,sink}\leftarrow GetRelativeDistance\left(\right)$;13:        $d_{i,j}\leftarrow CalculateRelativeDistance\left(\right)$;14:    **end if**15:    **if** $d_{i,sink}>d_{i,j}$ and $d_{i,sink}>d_{j,sink}$ and $d_{i,j}^{k}a{\left(f\right)}^{d_{i,j}}+d_{j,sink}^{k}a{\left(f\right)}^{d_{j,sink}}<d_{i,sink}^{k}a{\left(f\right)}^{d_{i,sink}}$ **then**16:        Neighbor.nodeID←j;17:        Neighbor.Q← Calculate the Q value according to the cost function (21);18:        Neighbor.EL←EL(j);19:        NbList.Add(Neighbor);20:    **else**21:        drop the packet;22:    **end if**23:**end procedure**

#### 4.2.2. Data Transmission Phase

In data transmission phase, the sensor nodes mainly have two operations: forwarding data packets and updating residual energy levels. The data packets in a node are either sensed by the node’s sensor or received from other nodes. The unique ID of the chosen forwarding node is included in data packet header and the receivers can check if it is eligible to forward the data packet. When a node is responsible for sending a data packet, it first checks its neighbor list and the sink node’s location. There are three cases in data forwarding process: (1) the EL of the sender is smaller than or equal to at least one of its neighbors’ EL; (2) the EL of the sender node is larger than that of all its neighbors and the sink node is within its maximum transmission range; (3) the EL of the sender is larger than that of all its neighbors and the sink node is located outside its maximum transmission range. In the first case, a subset of neighbors with larger or equal EL is constructed first. Then the neighborhood node with the minimal *Q* value in this subset is chosen as the next hop forwarder. In the second case, the data packet is just sent to the sink node directly. In the third case, a subset of neighbors with the largest EL is chosen first and then the node with the minimal *Q* in this subset is chosen as the next hop forwarder. After choosing the next hop node, the data packet header is refreshed with the updated node ID and data packet is sent to the chosen node. The detailed process of data forwarding is shown in Algorithm 2. The relative parameters are also defined in Table 1. After a period of time, the residual energy level may change and nodes need to inform their neighbors of their EL changes. Each time a node detects an EL change, it broadcasts an EL notice packet with its ID and EL. Then the receivers can update their neighbors’ EL according to the new received notice packet.
**Algorithm 2** Data Forwarding Process1:**procedure**SendData2:    $Node_{opt}.EL\leftarrow 0$;3:    $Node_{opt}.Q\leftarrow +\infty$;4:    $Node_{opt}.ID\leftarrow Null$;5:    **for** m←1 **to** $Num_{nb}$ **do**6:        **if** $Node_{opt}.EL<EL\left(i\right)$ **then**7:           **if** $Node_{opt}.EL<NbList\left(m\right).EL$ **then**8:               $Node_{opt}\leftarrow NbList\left(m\right)$;9:           **else if** $Node_{opt}.EL=NbList\left(m\right).EL$ and $NbList\left(m\right).Q<Node_{opt}.Q$ **then**10:               $Node_{opt}\leftarrow NbList\left(m\right)$;11:           **end if**12:        **else if** NbList(m).EL≥EL(i) and $NbList\left(m\right).Q<Node_{opt}.Q$ **then**13:           $Node_{opt}\leftarrow NbList\left(m\right)$;14:        **end if**15:    **end for**16:    **if** $Node_{opt}.EL<EL\left(i\right)$ and TR≥d(i,sink) **then**17:        $Node_{opt}.ID\leftarrow SinkID$;18:    **end if**19:    **if** $Node_{opt}.ID=Null$ **then**20:        drop the packet;21:    **else**22:        Update data packet header with $Node_{opt}.ID$;23:        Broadcast the packet;24:    **end if**25:**end procedure**

## 5. Simulations

**Figure 5:**
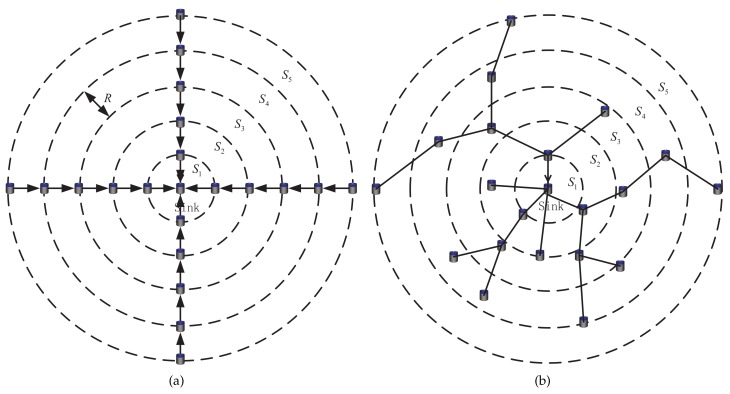
(**a**) regular node distribution, (**b**) random node distribution.

In this section, we present some simulation results to verify the effectiveness and validation of EBLE. The performance evaluation is done using the data from NS-3 simulator, which is a discrete event simulator for network simulation [32]. In this section, we conduct simulations on two kinds of node distribution as shown in Figure 5. Figure 5a is a regular node distribution case. In this case, the network is divided into *n* concentric circular rings S1,S2,S3…Sn and each ring’s radius is *R*. A sink node is deployed in the center of the network. The sensor nodes are deployed with equal spacing and each ring has the same number of sensor nodes. It is obviously seen that the node density is higher when the concentric circular rings are closer to the sink node. The sensor nodes that are closer to the sink have to transmit more data in multi-hop transmission mode as these nodes are required to relay more data loads to the sink. Figure 5b is a universal case in which the sensor nodes are deployed randomly in a circle with radius nR. The node density in each area of the network is a random value and the sensor nodes may not have more data loads when they are closer to the sink in multi-hop transmission mode. If the routing paths remain unchanged, some nodes may only need to send data packets that are created by themselves even when they are closer to the sink node. The relative simulation parameters are shown in Table 2. We value our proposed EBLE protocol against BEAR [16], BTM [17] and one-hop direct transmission in terms of energy consumption, average residual energy, average end-to-end delay and network lifetime. Here we define that a node is considered to be dead when it consumes 90% battery power of itself and the network lifetime is defined in two versions: the FirstNodeDead means the time when a first node is dead and the 50%Dead means the time when 50% of the sensor nodes are dead. When a node is dead, it broadcasts an energy depletion notice packet to its neighborhood nodes and the dead node cannot transmit any packets any more. In the following simulations, we first conduct simulations in the regular node distribution case and then in the randomly deployed case.

### 5.1. Regular Node Distribution

**Figure 6:**
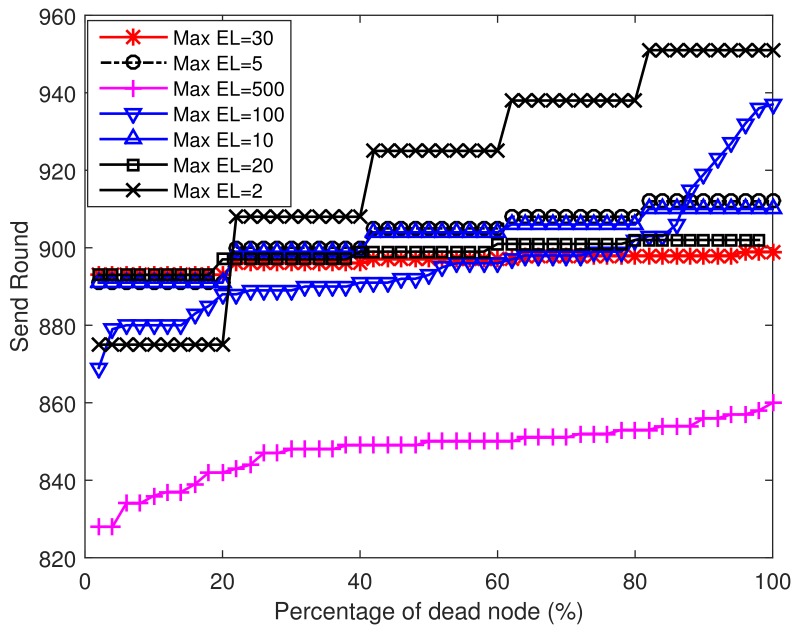
Percentage of dead nodes at different sending rounds.

In this part, we analyze the performance in the regular node distribution case. If not specified, the network is divided into five concentric circular rings and each ring contains four nodes. The radius *R* of each ring equals 500 m. Here we assume that each sensor node generates a data packet and forwards it to the sink in each round. A more realistic data load distribution will be analyzed in the next section. First, we discuss the effect of maximal EL to our proposed algorithm. Figure 6 presents the sending rounds when different percentages of sensor nodes deplete their battery power. We simulate seven cases and the maximal EL varies from 2 to 500. From this figure we can see that the maximal EL can affect the network life to some extent. When the maximal EL becomes very large (e.g., 500), the sensor nodes consume a lot of energy on broadcasting and upon receiving EL changes. Therefore, the sensor nodes are unable to send more data packets. However, when the maximal EL becomes very small (e.g., 2), the sensor nodes are unable to distinguish remaining energy changes among neighborhood nodes and a lot of energy is wasted on long range transmissions. When the first node in the network is dead, the network sends the largest number of data packets at the case of maximal EL = 20 and maximal EL = 30. In addition, in the case that maximal EL = 20 performs better after 20% of nodes in the network are dead when compared with the case of maximal EL = 30. So in the next simulations, we set maximal EL = 20. It should be noted that the energy consumption of broadcasting residual energy level information and determining relative distance makes up only a small proportion of the total energy consumption. The reasons are listed as follows:

- The network structure we consider here is a quasi-stationary one which means the sensor nodes rarely move after deployment. So nodes need to broadcast beacon messages only once to obtain the relative distance towards the sink node and their neighborhood nodes. In addition, the cost of broadcasting beacon messages becomes relatively low when comparing with continuous data transmissions.
- Although each sensor node needs to broadcast its EL changes 19 times (maximal EL = 20) before it depletes all its energy, the frame length of beacon messages and EL change notice packet is small as these control packets have no data load. So the total energy consumption for transmitting control packets is low.

In order to further present the cost for control packets, we calculate the percentage of energy consumption for different transmitting phases. In this regular node distribution case, the result shows that the sum of energy consumption in broadcasting/receiving EL notice packets and beacon packets makes up 3.48% of the total energy consumption (maximal EL = 20). In addition, this small part of energy consumption can prolong the network lifetime significantly by energy balancing.

**Figure 7:**
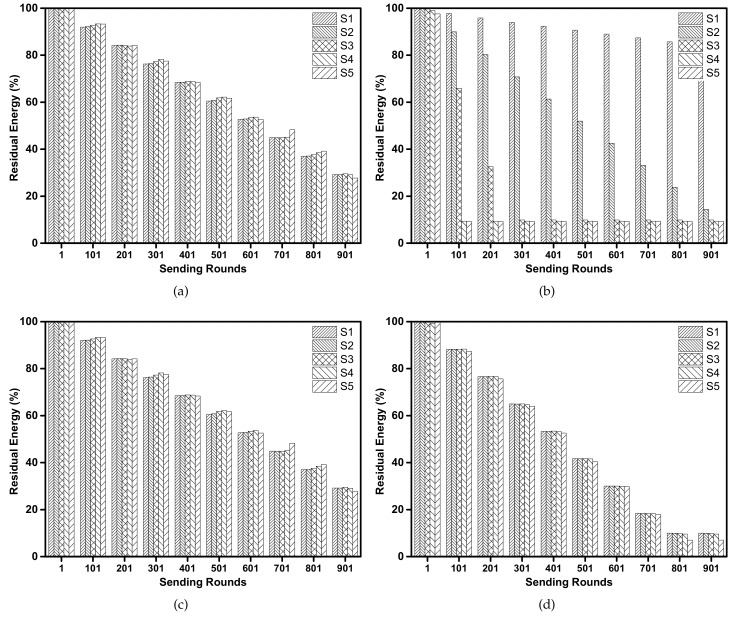
Percentage of residual energy: (**a**) EBLE, (**b**) Direct transmission, (**c**) BTM, (**d**) BEAR.

Figure 7 shows the percentage of node residual energy at different sending rounds for different protocols. It can be seen that the direct one-hop transmission protocol (Figure 7b) is not suitable for long time transmissions because the sensor nodes far away from the sink consume their battery power at a high speed. When the sensor nodes in S5 are dead, the sensor nodes in S1 still have over 90% battery power. At sending round 901, about 75% of sensor nodes are dead and cannot transmit data packets any more. In BEAR protocol (Figure 7d), the energy consumptions among sensor nodes in different circular rings are similar due to the use of energy balancing scheme. However, the overall energy consumption of BEAR is high as all sensor nodes are dead after 801 sending round. This is because in BEAR, each sensor node needs to broadcast control packets after each sending round to inform its neighbors of its residual energy. This operation wastes a significant amount of energy. In Figure 7a,c, the BTM protocol and our proposed EBLE show a similar improvement in terms of energy consumption when compared with BEAR and direct one-hop transmission. All sensor nodes are kept alive at the 901 sending round and nodes in different circular rings consume their battery power at a similar speed. Although EBLE use more candidate forwarding nodes than BTM, there are no significant gaps in the energy consumption performance. This is because the nodes in the network are distributed regularly, which means the optimal energy efficient paths for multi-hop transmission are the same for BTM and EBLE. When the next candidate forwarder’s EL is lower than the sender, the sender cannot find other candidate sensor nodes which have more energy and are closer to the sink. So both BTM and EBLE change their transmission mode to direct one-hop transmission when their forwarders lack energy.

**Figure 8:**
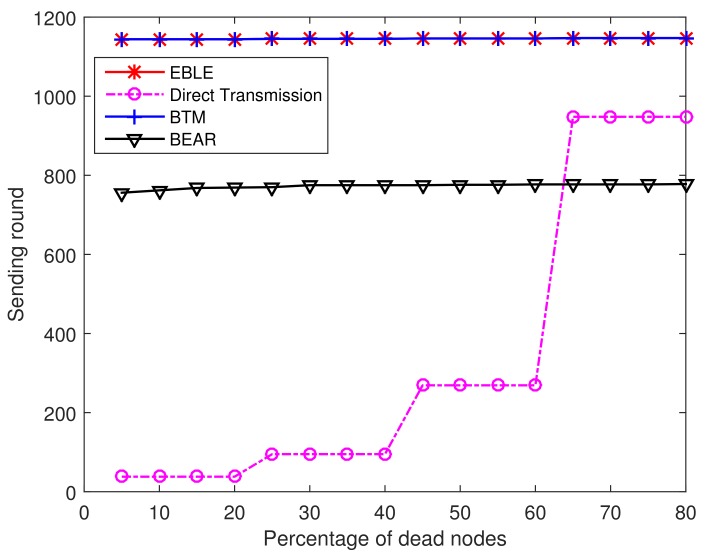
Percentage of dead nodes at different sending rounds.

Figure 8 illustrates the percentage of dead nodes in varying sending rounds. For direct transmissions, 20% of the sensor nodes are dead at the 38th sending round and 70% of the sensor nodes are dead before the 270th sending round. So it is clearly seen that the direct one-hop transmission protocol cannot use the battery power in a balanced way. For BEAR, the energy consumption among different nodes are balanced and almost all sensor nodes are dead at the same time. However, BEAR uses part of its battery power to broadcast its residual energy information and makes the network lifetime end at an early time. BTM and EBLE achieve better performance in the regular node distribution case as the sensor nodes can transmit more data packets before their battery power is depleted.

**Figure 9:**
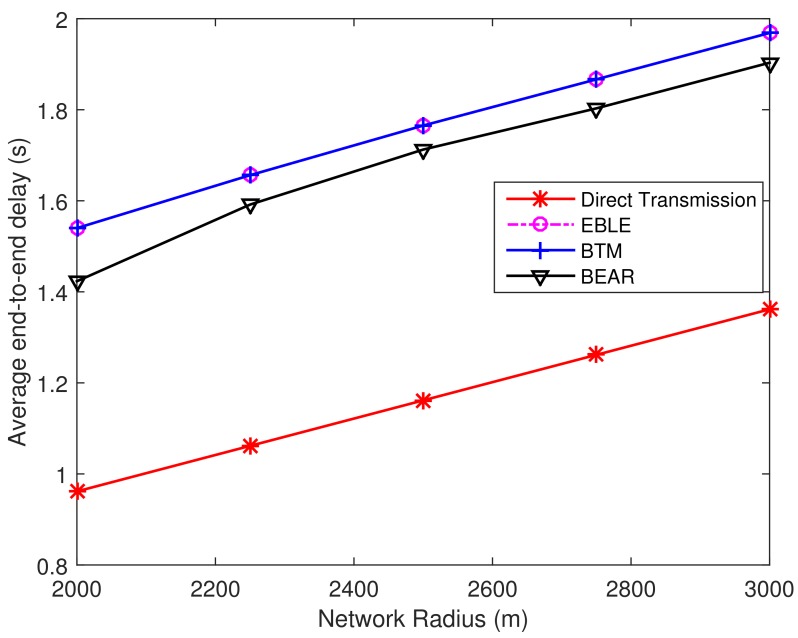
Average end-to-end delay. The network radius is 1500 m.

Figure 9 shows the average end-to-end delay for the four protocols. The communication delay for direct one-hop transmissions is the lowest because data packets are forwarded to the sink directly without any relay. Here we assume that each node spends 0.1 s to process the data and state transition (from receiving state to transmitting state). So each relay induces at least 0.1s delay for each sending process. The delay also comes from possible longer routing paths. This part of the delay cannot be avoided for multi-hop transmissions. So EBLE/BTM is slightly inefficient in network throughput, but the network lifetime is prolonged significantly for EBLE and BTM.

**Figure 10:**
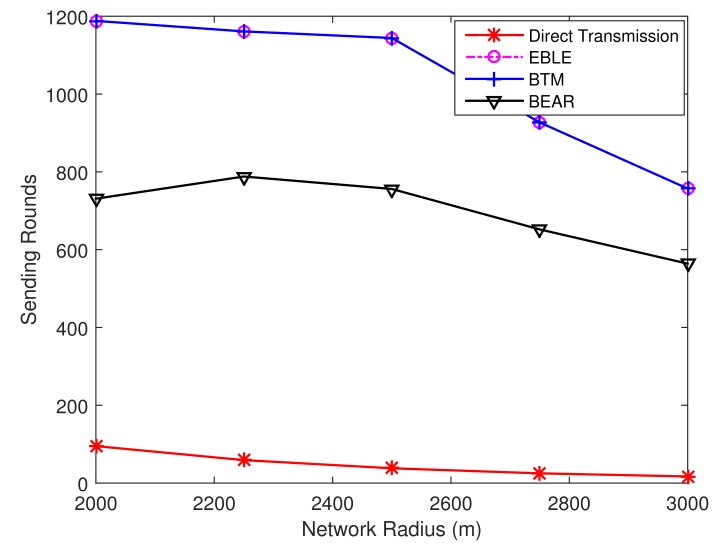
The sending round when a first node in the network is dead.

**Figure 11:**
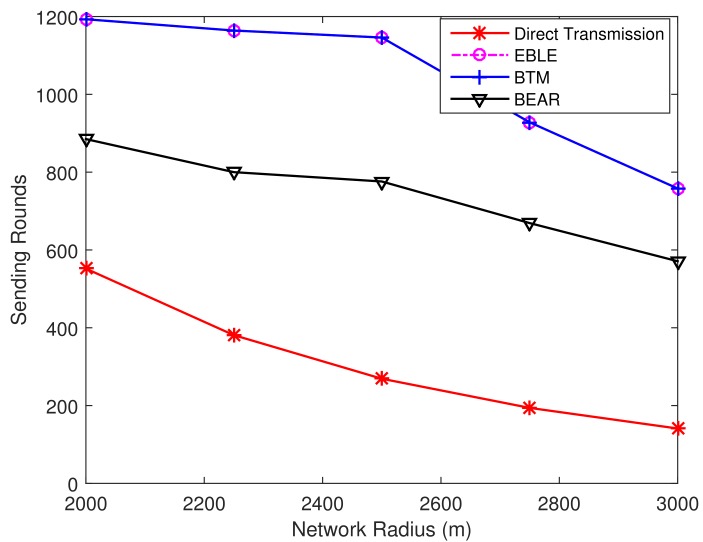
The sending round when half of the sensor nodes in the network are dead.

Figure 10 and Figure 11 illustrate the FirstNodeDead and 50%Dead network lifetime performance, respectively. It can be seen that the time points when the first node is dead and 50% node are dead are the same for EBLE and BTM. However, parts of the sensor nodes in direct transmission mode deplete their battery power at an early time, which makes the network unable to sense some parts in the distribution area. So the network ends much earlier than EBLE. The lifetime performance of BEAR protocol improves compared with direct one-hop transmission protocol, but it still performs significantly worse than EBLE and BTM.

**Figure 12:**
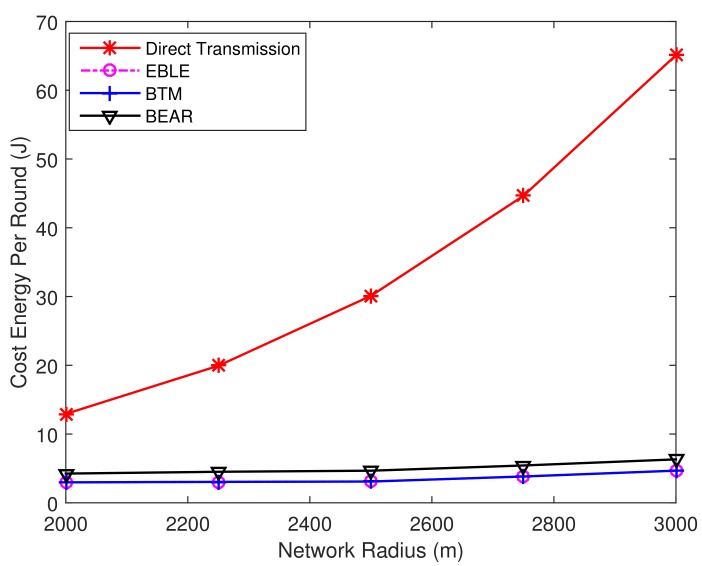
The overall network consumed energy per sending round for different protocols.

Figure 12 shows the overall network consumed energy per sending round for different protocols. The energy consumption for direct transmissions is high and the performance is even worse for long network radius. EBLE, BTM and BEAR utilize multi-hop transmissions to make the network more energy efficient. The average network consumed energy per sending round does not vary significantly with increasing network radius; EBLE is more energy efficient than BEAR and direct transmission. The performance of EBLE and BTM seems the same for the regular node distribution case.

### 5.2. Random Node Distribution

In this part, we value the performance among different protocols under random node distribution as shown in Figure 5b. At each sending round, each node generates *X* data packets randomly using Possion distribution. The probability distribution function is given in Equation (22). Here we set λ=0.5, so each node send 0.5 packets on average per sending round. The number of sensor nodes are 40 and the network radius is 2 km.

(22) $$P\left(X=k\right)=\frac{\lambda^{k}}{k!}e^{-\lambda },k=0,1,\cdots$$

**Figure 13:**
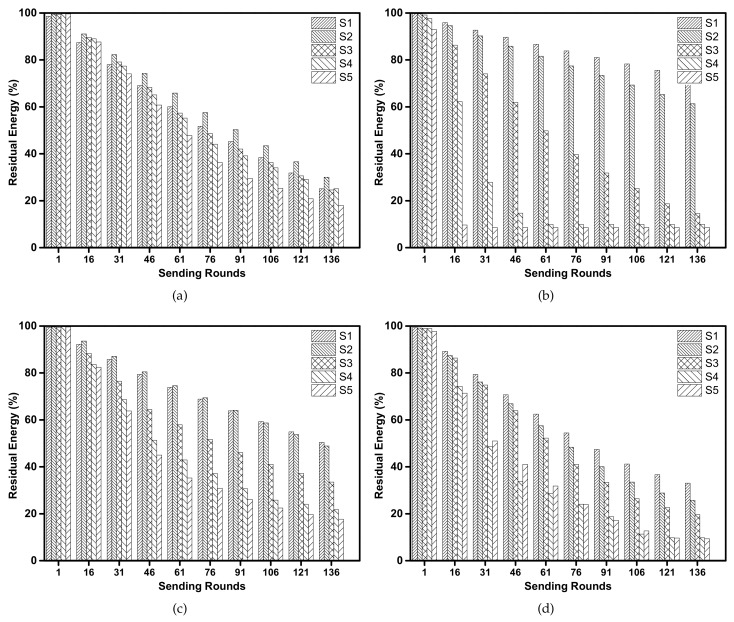
Percentage of residual energy: (**a**) EBLE, (**b**) Direct transmission, (**c**) BTM, (**d**) BEAR.

Figure 13 shows the percentage of residual energy at different circular rings for EBLE, direct transmission, BTM and BEAR. There is a significant difference in energy consumption at different rings for direct transmission protocol (Figure 13b). Both BTM and BEAR improve the unbalanced energy consumption to some extent but the difference is still large. As the sensor nodes are not deployed evenly, nodes in some areas may deal with heavy data loads and BTM/BEAR fails to balance the energy consumption between these areas. Our proposed EBLE outperforms other protocols in terms of energy balancing. This is because that EBLE can balance the energy consumption with more candidate forwarding nodes. Once the most energy efficient path is energy limited, a suboptimal route can be chosen and the direct one-hop transmission only happens when all candidate forwarding nodes lack battery power.

**Figure 14:**
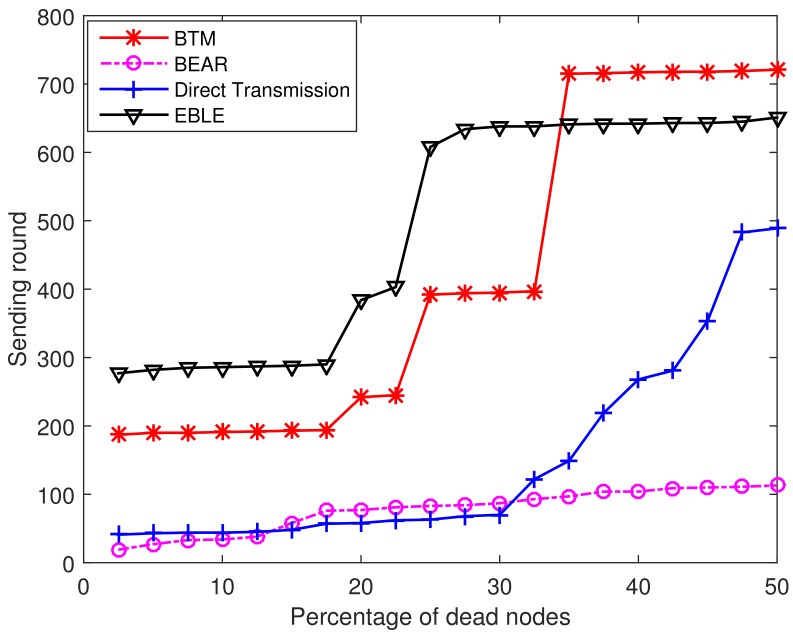
Percentage of dead nodes at different sending rounds.

Figure 14 shows the percentage of dead nodes at different sending round for direct transmission, EBLE, BTM and BEAR. In this random node distribution case, EBLE can send the greatest number of packets before a first node in the network is dead. When a first node is dead because of low power, EBLE can have 90 more sending rounds than BTM. The performance of BEAR is even worse than the direct transmission in this case. This is because BEAR uses regular zones to choose the next forwarder. Only nodes in the same zone can be selected as next hop forwarders and this limits the choice of forwarders. When the nodes in the network are distributed randomly, there may not be enough nodes in some zones and BEAR has to choose to send data to the sink node directly. The energy cost for broadcasting residual energy also aggravates the energy consumption. It can also be seen that when 35% of the sensor nodes are dead, BTM can send more packets compared with EBLE. This is because BTM uses more direct transmissions than EBLE and some nodes in BTM may have few data loads, which makes the network keep a lot of energy when some nodes are unable to work any more. In network protocol design, we believe that the principle is to keep as many sensor nodes alive as possible because we are unwilling to lose the information of any interested area.

**Figure 15:**
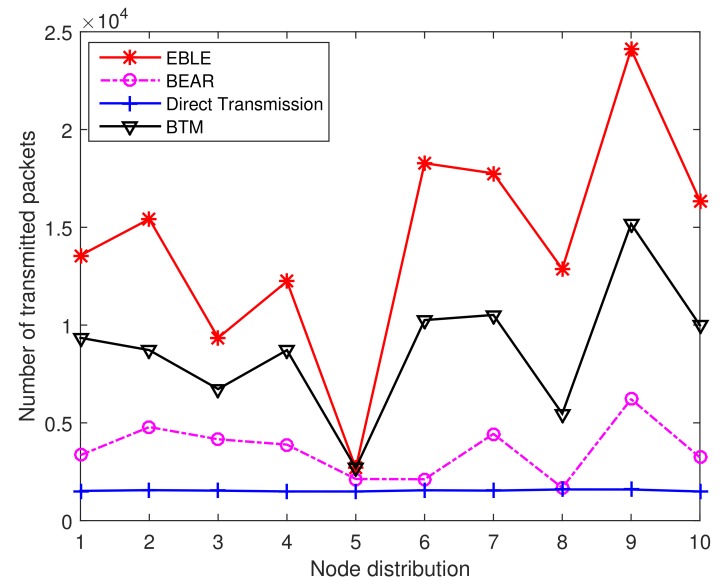
The number of transmitted packets for different distributions when a first node is dead.

In order to make the simulation results more reliable, we change the random seed to conduct simulations on 10 different random node distributions. Figure 15 shows the number of overall transmitted packets in the network when a first node is depleted of its battery power. The direct one-hop transmission protocol has almost the same number of transmitted packets over different node distributions. This is because the network life performance for direct transmission is always determined by the node farthest from the sink. Other protocols use energy balancing scheme to distribute the data load evenly. It is clearly seen that EBLE outperforms other protocols in terms of FirstNodeDead network life. This is mainly due to our proper design of cost function and intelligent choice of next forwarders. In BTM, only one node has a unique optimal forwarder. If this forwarder is in low battery power state, BTM has to send data directly to the sink. However, EBLE selects the next hop forwarders by comparing their energy consumption per effective distance. The node with the lowest energy consumption per effective distance is chosen as the best forwarder. When the residual energy of the best forwarder is relatively low, EBLE selects the suboptimal node with higher residual energy for relaying data instead of long range one-hop direct transmission to the sink node. So BTM performs worse than our proposed EBLE in the random node distribution case. From the 10 groups of results in Figure 15, we can calculate that EBLE can send 62.79% more packets than BTM on average before a first node is dead.

**Figure 16:**
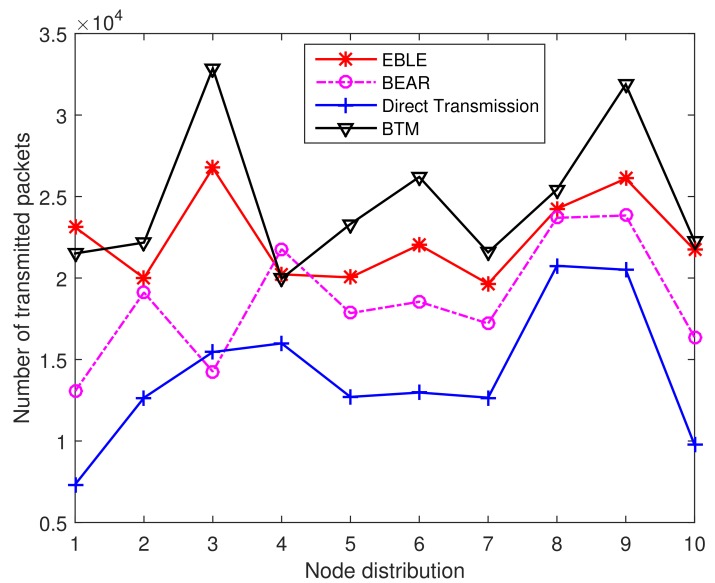
The number of transmitted packets for different distributions when 50% nodes are dead.

To explain the performance fully, we present the 50%Dead network lifetime performance as shown in Figure 16. In this case, EBLE no longer performs better than BTM. This is because EBLE balances the network energy consumption to ensure that all the sensor nodes can work. In BTM, some nodes may only have a few data loads, therefore these nodes can still work for a long time when other nodes are dead. We believe that this unbalanced energy consumption is unhealthy for underwater sensor networks. So we mainly focus on the optimization of FirstNodeDead network life performance.

**Figure 17:**
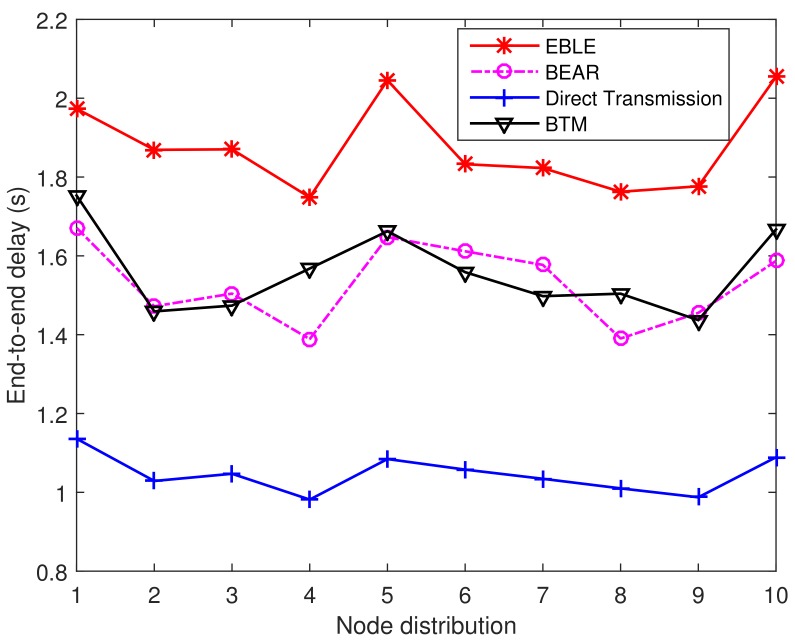
The average end-to-end delay per transmitted packet for different distributions.

Next, we consider the end-to-end delay performance as shown in Figure 17. It can be seen that the average time for transmitting one packet in EBLE is prolonged. This is because EBLE is more likely to divide long range direct transmissions into multi-hop transmissions. In our settings, each relay needs 0.1 s to handle the received data packets and change its transducer state from receiving mode to transmitting mode. So the more relays that are used, the longer time a data transmitting process needs. The irregular paths also induce some propagation delay to some extent. So our design may not be suitable for some delay sensitive applications. However, as the induced delay is not significant and is about 1.8 times that of the direct transmissions, EBLE can be used in most long time data transmission cases.

**Figure 18:**
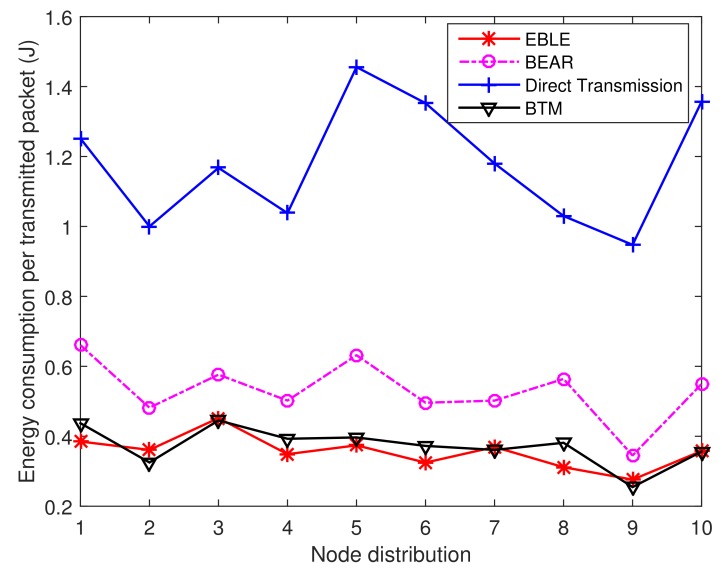
The average energy consumption per transmitted packet for different distributions.

The energy consumption per transmitted packet for different protocols is given in Figure 18. The energy consumption for direct transmission protocol is much higher than other protocols because long-range direct transmissions are not energy efficient and the sender has to send data at a higher power to reach the sink node far away. The performance of BTM and EBLE is similar and they are both better than BEAR and direct transmission. This is because EBLE selects the next hop forwarders by comparing their energy consumption per effective distance. The node with the lowest energy consumption per effective distance is chosen as the best forwarder. So long range direct transmissions can be divided into multi-hop transmissions effectively.

## 6. Conclusions

In this paper, we propose EBLE routing protocol for UWSNs. EBLE exploits the location and residual energy level information to select candidate next-hop forwarders. Energy efficiency and energy balancing are both considered in cost function design and forwarder selection process. Then the nodes with low energy cost and relatively high energy level are chosen as the optimal forwarder. The simulations are conducted in two cases: regular node distribution case and random node distribution case. The results show that the proposed EBLE can balance the network energy consumption and prolong the network lifetime in both cases. In the regular node distribution case, EBLE achieves the same performance as BTM in terms of network lifetime and energy efficiency, which is much better than BEAR and direct transmissions. In random node distribution case, EBLE can send 62.79% more packets than BTM on average before a first node is dead. We can also see that EBLE improves the energy efficiency at the cost of introducing some delay. So EBLE can be used in energy-limited and delay-insensitive applications.

## Figures and Tables

**Table 1:** Nomenclature.

Notation	Definition
$Node_{opt}$	chosen forwarding node register
EL(i)	residual energy level of current node *i*
EL(j)	residual energy level of sending node *j*
Neighbor	neighbor node register
$Num_{nb}$	number of stored neighbor nodes
NbList	stored neighbor list
TR	maximum transmission range

**Table 2:** Simulation Settings.

Parameter	Value
Data Rate	10 kbps
Center Frequency	20 kHz
Bandwidth	10 kHz
Receiving Power	0.03 W
Transmitting Power	0.17–29 W
Mode Type	FSK
Packet Error Rate Model	ns3::UanPhyPerDefault
Signal Noise Model	ns3::UanPhyCalcSinrDefault
Acoustic Propagation Speed	1500 m/s
UAN Propagation Model	ns3::UanPropModelThorp
MAC Model	CWMAC
Required SNR for Signal Acquisition	20 dB reμPa
Payload of DATA	200 Bytes
Network Radius	1–2 km
Initial Energy per Node	200 J
Node Number	20–50
Number of Concentric Circular Rings	5
